# Interpersonal Violence during the COVID-19 Lockdown Period in Nepal: A Descriptive Cross-sectional Study

**DOI:** 10.31729/jnma.5499

**Published:** 2020-10-31

**Authors:** Calvin Ghimire, Sajan Acharya, Carmina Shrestha, Prabhat KC, Swarndeep Singh, Pawan Sharma

**Affiliations:** 1 Patan Academy of Health Sciences-School of Medicine, Lalitpur, Nepal; 2 New York Medical College/Metropolitan Hospital Center, New York, USA; 3 Department of Psychiatry, All India Institute of Medical Sciences, New Delhi, India; 4 Department of Psychiatry, Patan Academy of Health Sciences-School of Medicine, Lalitpur, Nepal

**Keywords:** *COVID-19*, *domestic violence*, *interpersonal violence*, *Nepal*, *substance abuse*

## Abstract

**Introduction:**

The government issued a country-wide lockdown in Nepal as a measure to curb the spread of COVID-19 pandemic. This has resulted in various difficult experiences which includes financial loss, separation from loved ones, grief, uncertainty over disease status and loss of freedom. During these stressful situations, interpersonal violence is likely to be aggravated. To avoid the occurrence of adverse events such as impulsive acts, homicide, or suicide, it is important to identify high-risk individuals.

**Methods:**

This is a descriptive cross-sectional, questionnaire-based, online survey by convenience sampling. The prevalence of different types of interpersonal violence with socio-demographic factors, substance use, and overall mental wellbeing was assessed by using descriptive statistical tests.

**Results:**

Out of total 556 participants included in the analysis, 50.9% (283) were male and 48.7% (271) were female. There were 100 (18.0%) participants who reported being a victim of interpersonal violence and 101 (18.2%) participants who reported being a perpetrator during the lockdown. The victims of violence were more likely to be living with their spouse alone. The victims and perpetrators were also more likely to have increased alcohol and tobacco use. More number of victims and perpetrators had lower mental wellbeing scores on the WHO wellbeing index.

**Conclusions:**

There was prevalence of interpersonal violence during the COVID-19 lockdown. In addition to the fear regarding pandemic, victims have to face domestic violence placing them at a double injustice. Identification of vulnerable groups and proper management of survivors must be prioritized given the unanimous consensus on the rise of interpersonal violence during periods of heightened stress.

## INTRODUCTION

Nepal was on country-wide lockdown starting from 24 March 2020 as a measure imposed by the government to curb the novel coronavirus virus disease 2019 (COVID-19). The lockdown period was extended in increments and ended on 21 July 2020.^1^ This measure to contain and manage the pandemic has given rise to a pattern of adverse experiences such as loss of employment, financial hardship, social isolation, grief, school closures, and property loss.^2^

During stressful situations faced during the pandemic, violence of all etiologies in domestic settings are likely to rise.^2^ Deviant behaviors towards children due to difficulties in managing parental stress are likely to rise as well.^3^

Identification of people at high risk of violence, impulsive acts, homicide, and suicide is extremely important to ensure good mental health during lockdown. The present study aimed to explore the pattern of interpersonal violence experienced by people living in Nepal during the lockdown period.

## METHODS

This was an open descriptive, web-based, cross-sectional study designed following the CHERRIES checklist.^4^ The study was approved by the institutional review committee of Nepal Health Research Council (Ref: 332/220p). The study was conducted for a period of 4 months (April 1st to July 30th, 2020). The questionnaire included demographic variables and the World Health Organization wellbeing Index (WHO-5)^5^ questions to assess subjective wellbeing. The WHO-5 is a 5-item questionnaire which segregates participants with a score below or equal to 13 as having poor mental wellbeing, requiring further evaluation for depression. Information regarding domestic violence and substance abuse were collected using questions designed by the study team consisting of medical doctors and psychiatrists. The questions were included on the basis of face validity. The questionnaire was pretested among a group of 15 participants to ensure clarity and validity. The pre-test participants were excluded from the study.

Convenience sampling was used with the web-based survey link sent in various social media groups (Facebook, Linkedin, Twitter) using specifically created social media accounts. The link to the survey was shared multiple times with no benefits from participation and purpose of study clearly mentioned. Informed consent from participants was obtained in the same google form used for the study. The form only contained the initials and credentials of the investigators and no identifiers. Storage of data was done using password-protected google drive with investigator limited access. To ensure that participants were still living inside the country, they were asked to provide the name of the city they resided during the lockdown. Participants living outside Nepal were excluded. Incomplete questionnaires were not used in the analysis. The completion rate was 0.986, 556/564. Participants were only allowed to change their answers before clicking submit. The participants were requested to only fill the form once and close the link if they have filled it previously, to prevent repetition of study samples.

The data were analyzed using SPSS version 23.0 (Armonk, NY, IBM Corp). Descriptive statistics using mean, standard deviation, frequency and percentage were used to describe the socio-demographic profile, substance use pattern, pattern of interpersonal violence, and the WHO wellbeing index scores of the study participants.

## RESULTS

A total of 556 participants were included in the final analysis. There were 283 (50.9%) male and 271 (48.7%) female participants. Two participants chose not to disclose their gender. The mean age of the study participants was 25.93 ± 6.88 years. There were 110 (19.8%) married, 2 legally separated, 1 divorced and 1 widowed participants. All the study participants had received some form of formal education. The current living arrangement reported by study participants during the lockdown period varied from being alone 57 (10.3%), with spouse only 16 (2.9%), with family 422 (75.9%), with friends 44 (7.9%), with other relatives 12 (2.2%) and with others 5 (0.9%).

When asked if violence has occurred to them during the lockdown, 11 participants chose the “prefer not to say” option. Also, 10 participants chose the “prefer not to say option” when asked if they had committed violence during the lockdown. The participants who chose the “prefer not to say” option were counted as having experienced or as having committed violence during lockdown accordingly.

A total of 100 (18.0%) participants reported being a victim of Interpersonal Violence (IPV) during the lockdown. Among them, 49 (49%) experienced verbal violence only, 4 (4%) experienced physical violence only while 47 (47%) experienced both. Participants of lower age-groups were more likely to face violence. A slightly higher number of males (51, 18%) reported being a victim of IPV compared to females (47, 17.3%). Higher number of participants who had never married (85, 19.2%) experienced IPV versus those who were married (15, 13.6%). Participants with lower education level were more likely to be a victim of IPV (Table 1). Participants living with a spouse alone (6, 37.5%) were most likely to experience violence followed by those living with friends (13, 29.5%).

**Table 1:** Socio-demographic profile of victims and perpetrators of interpersonal violence (n = 556).

Variables	Total Participants n (%)	Victim of IPV^*^ n (%)	Perpetrator of IPV^†^ n (%)
**Age**			
18 to 23	231 (41.5)	54 (23.4)	43 (18.6)
24 to 29	214 (38.5)	32 (15.0)	38 (17.8)
30 to 35	83 (14.9)	9 (10.8)	13 (15.7)
> 36	28 (5.0)	5 (17.9)	7 (25.0)
**Gender**			
Female	271 (48.7)	47 (17.3)	49 (18.1)
Male	283 (50.9)	51 (18.0)	50 (17.7)
Prefer not to say	2 (0.4)	2 (100.0)	2 (100.0)
**Marital Status**^‡^			
Married	110 (19.8)	15 (13.6)	21 (19.1)
Never married	442 (79.5)	85 (19.2)	80 (18.1)
**Education**			
10 + 2 or equivalent	124 (22.3)	33 (26.6)	22 (17.7)
Bachelor’s degree	304 (54.7)	52 (17.1)	58 (19.1)
Master’s degree or above	128 (23.0)	15 (11.7)	21 (16.4)
**Living Arrangement**			
Alone	57 (10.3)	7 (12.3)	4 (7.0)
With family	422 (75.9)	71 (16.8)	74 (17.5)
With friends	44 (7.9)	13 (29.5)	15 (34.1)
With relatives	12 (2.2)	2 (16.7)	1 (8.3)
With spouse only	16 (2.9)	6 (37.5)	6 (37.5)
Others	5 (0.9)	1 (20.0)	1 (20.0)

* 11 victims chose the “prefer not to say” option when asked if they had experienced violence during the lockdown.

† 10 perpetrators chose the “prefer not to say” option when asked if they had been a perpetrator of violence during the lockdown.

‡ Two participants reported living as legally separated couples, one participant reported to be divorced, and another one to be widowed.

There were 101 (18.2%) participants who reported being a perpetrator of violence during the lockdown. Among them, 43 (42.6%) reported being a perpetrator of verbal violence only, 9 (8.9%) reported having carried out physical violence only while 49 (48.5%) reported having committed both. Older participants more than or equal to 36 years of age (7, 25%) were more likely to report being a perpetrator of IPV followed by participants who were 18 to 25 years of age (43, 18.6%), 24 to 29 years of age (38, 17.8%) and 30 to 35 years of age (13, 15.7%). Prevalence of reporting being a perpetrator of IPV was similar among both males (50, 17.7%) and females (49, 18.1%). Also, both married (21, 19.1%) and never married (80, 18.1%) were similarly likely to commit IPV. Participants holding a master's degree and above (21, 16.4%) were least likely to commit IPV. Those living with spouse alone (6, 37.5%) were most likely to report being a perpetrator of IPV followed by participants living with friends (15, 34.1%) (Table 1).

Participants with a previously diagnosed mental disorder were more likely to report being a victim (12, 27.3%) as well as a perpetrator (11, 25%) of IPV. Poor mental wellbeing as indicated by WHO-5 score less than equal to 13 was more prevalent among both victim (63, 28.3%) and perpetrator (54, 24.2%) of IPV. During the lockdown, 13 (2.3%) participants reported an increase in their tobacco smoking and 17 (3.1%) of the participants reported an increase in their alcohol consumption. More participants who had experienced IPV (6, 46.2%) and those who had committed IPV (5, 38.5%) reported an increase in their tobacco consumption during lockdown than those who had decreased or no change in tobacco use pattern. Similarly, those who had experienced IPV (4, 23.5%) and those who had committed IPV (5, 29.4%) reported an increased alcohol intake during lockdown compared to those who had decreased or no change in alcohol use (Table 2).

**Table 2:** Mental wellbeing and substance use among victims and perpetrators of interpersonal violence (n = 556).

Variables	Total Participants n(%)	Victim of IPV ^*^ n(%)	Perpetrator of IPV ^†^ n(%)
**Mental Disorder**			
No	512 (92.1)	88 (17.2)	90 (17.6)
Yes	44 (7.9)	12 (27.3)	11 (25.0)
**Mental Wellbeing**			
≤13	223 (40.1)	63 (28.3)	54 (24.2)
>13	333 (59.9)	37 (11.1)	47 (14.1)
**Smoking**			
Decreased	84 (15.1)	13 (15.5)	14 (16.7)
No change	459 (82.6)	81 (17.6)	82 (17.9)
Increased	13 (2.3)	6 (46.2)	5 (38.5)
**Drinking**			
Decreased	150 (27.0)	23 (15.3)	25 (16.7)
No change	389 (70.0)	73 (18.8)	71 (18.3)
Increased	17 (3.1)	4 (23.5)	5 (29.4)

* 11 victims chose the “prefer not to say” option when asked if they had experienced violence during the lockdown.

† 10 perpetrators chose the “prefer not to say” option when asked if they had been a perpetrator of violence during the lockdown.

According to victims, siblings (18, 18%) were the most likely perpetrators of interpersonal violence followed by parents (17, 17%). Sixteen (16%) participants reported being victimized by multiple perpetrators. According to the type of violence, siblings (10, 10%) were most likely to commit both physical and verbal violence followed by friends (9, 9%). The detailed description of different types of interpersonal violence and perpetrators reported by the study participants has been provided (Table 3).

**Table 3:** Perpetrators of interpersonal violence during lockdown according to the victim (n = 100).

Perpetrators of IPV according to victim	Total Perpetrators n (%)	Type of Violence		
		Physical Only n (%)	Verbal Only n (%)	Both Physical and Verbal n (%)
Children	1 (1.0)	-	-	1 (1.0)
Siblings	18 (18.0)	2 (2.0)	6 (6.0)	10 (10.0)
Spouse	6 (6.0)	-	5 (5.0)	1 (1.0)
Romantic partner	7 (7.0)	-	3 (3.0)	4 (4.0)
Parents	17 (17.0)	-	10 (10.0)	7 (7.0)
Friends	11 (11.0)	-	2 (2.0)	9 (9.0)
Others	4 (4.0)	-	4 (4.0)	-
Multiple perpetrators	16 (16.0)	1 (1.0)	10 (10.0)	5 (5.0)
Prefer not to say	20 (20.0)	1 (1.0)^*^	9 (9.0)^†^	10 (10.0)^‡^

* 1 victims chose the “prefer not to say” option when asked if they had experienced physical violence during the lockdown.

† 7 victims chose the “prefer not to say” option when asked if they had experienced verbal violence during the lockdown.

‡ 3 victims chose the “prefer not to say” option when asked if they had experienced physical and verbal violence during the lockdown.

According to perpetrators of IPV, siblings (26, 25.7%) were their most likely victims. Fifteen (14.9%) participants reported having victimized multiple people. According to the type of violence, siblings (13, 12.9%) were most likely to become victims of both physical and verbal violence followed by parents (9, 8.9%). The detailed description of different types of interpersonal violence and victims reported by the study participants has been provided (Table 4).

**Table 4:** Victims of interpersonal violence during lockdown according to the perpetrator (n = 101).

Victims of IPV according to perpetrators	Total victims n (%)	Type of Violence		
		Physical Only n (%)	Verbal Only n (%)	Both Physical and Verbal n (%)
Childrens	7 (6.9)	1 (1.0)	1 (1.0)	5 (5.0)
Siblings	26 (25.7)	4 (4.0)	9 (8.9)	13 (12.9)
Spouse	10 (9.9)	1 (1.0)	7 (6.9)	2 (2.0)
Romantic partner	6 (5.9)	-	4 (4.0)	2 (2.0)
Parents	14 (13.9)	-	5 (5.0)	9 (8.9)
Friends	7 (6.9)	-	2 (2.0)	5 (5.0)
Others	2 (2.0)	-	2 (2.0)	-
Multiple victims	15 (14.9)	1 (1.0)	10 (9.9)	4 (4.0)
Prefer not to say	14 (13.9)	2 (2.0)^*^	3 (3.0)^†^	9 (8.9)^‡^

* 2 perpetrators chose the “prefer not to say” option when asked if they had been a perpetrator of violence during the lockdown.

† 2 perpetrators chose the “prefer not to say” option when asked if they had been a perpetrator of violence during the lockdown.

‡ 6 perpetrators chose the “prefer not to say” option when asked if they had been a perpetrator of violence during the lockdown.

## DISCUSSION

Interpersonal violence refers to violence between individuals and is subdivided into family and intimate partner violence and community violence. Interpersonal violence can take the form of physical, sexual, psychological and can involve deprivation and neglect.^6^ There is a tendency for cases of domestic violence to increase following catastrophic events and rise in unemployment rates.^7^ Following the Cyclone Nargis in Myanmar, an increase by 30% in domestic violence was observed.^8^ Similar findings were reported post hurricane Katrina with a 35% surge of partner violence.^9^ In Uganda, the cases of domestic violence regularly spike following periods of drought.^10^

An alarming rise in cases regarding domestic violence have been reported worldwide since the initiation of isolation measures to curb COVID-19. Surges in calls in helpline numbers have been observed. The National Women Commision (NWC), Nepal which operates a 24-hour helpline for domestic violence survivors reported 1267 calls of domestic violence and 306 calls of violence against women during the 4 months lockdown period - a sharp rise from the period before lockdown. Out of these, 232 as cases were referred to appropriate partner organizations for immediate help.^11^

Countries in Europe also reported up to 60% rise in emergency calls compared to last year from women subjected to intimate partner violence.^12^ In Wuhan China, the domestic violence complaints received by a police station tripled compared with the same period last year.^13^ Many countries like France, Brazil, United States have reported a rise in number of cases of domestic violence during this pandemic, indicating a surge of cases worldwide.^14^

In our study, the frequency of verbal violence reported by the victim as well as perpetrator was higher compared to reported physical violence. Similar finding was found from data collected through helpline calls placed to NWC, where psychological torture was the most common violence followed by physical assault, economic and sexual violence. The same study also reported 71% of the perpetrators to be intimate partners or spouses.^11^ This finding also resonates with our study which demonstrated that victims of physical violence were more likely to be living with a spouse only. Participants living alone or with family revealed to be least likely to face violence.

Participants of younger age were found to be more likely to face violence in our study. However, our study was limited to participants above 18 years of age and the mean age of participants was 25.93 ± 6.88 years. More study including a wider age-range of participants is necessary to come to conclusions regarding the relationship between age of victim and frequency of violence during lockdown. NWC reported that 47% of the survivors of reported violence during lockdown were between 26 to 40 years of age.^11^

With restrictions on mobility and stay at home orders, victims of domestic violence are forced to remain with their perpetrators and also face difficulty reaching out for help. Increasing duration of COVID-19 lockdown and periodic extensions have added to the stress of coping with uncertainty, fear of disease, reduced income, confinement and lack of social support. The cases of violence at home, workplace and community are also likely to follow the compound.^12^ In our study more numbers of both the victim and the perpetrator of physical and verbal violence were found to have low WHO-5 wellbeing scores compared to those who did not face or commit violence. This indicates that the WHO-5 tool can potentially be used to help identify vulnerable populations likely to face violence. However, more studies on a larger scale is necessary to determine this relationship.

A surge in alcohol sale has been observed during this lockdown period in different countries.^15^ Experts have also warned against the harms of drinking at home.^16^ However, a survey done in the UK showed that while 21% participants had been drinking more frequently, 35% participants had reduced their alcohol intake.^17^ This finding is consistent with our study which revealed that a greater proportion of participants had decreased frequency of both alcohol consumption and smoking compared to those who had increased frequency. A study by Klemperar et al. attributed the increased use of tobacco and electronic cigarettes among previous users to be due to newly developed stressors during COVID-19.^18^ Spike in frequency of home drinking and cigarette smoking is likely to have a direct adverse impact on family members.^16,19^ Our study revealed an increased prevalence of drinking and smoking among the victims of physical violence as well as perpetrators of physical and verbal violence - demonstrating both cause and effect relationship.

Undergoing intimate partner violence increases the risk of multiple mental health conditions (mood disorders, anxiety disorders, eating disorders, posttraumatic stress disorder, substance or alcohol abuse) and physical health conditions (cardiovascular disease, chronic pain, sleep disturbances, gastrointestinal problems, sexually transmitted infections, traumatic brain injury).^20^ Children and adolescents exposed to violence (witnessing or victimization) by adults within the family, can develop anxiety, depression, eating disorders, substance use, self-harm or suicidal ideation, and poorer general health.^21^

This study has its own set of limitations. The study sample was small. More studies with large sample size would truly visualize the impact of COVID-19 on interpersonal violence, especially the magnitude of increment. The research was limited to internet users only. The study was time-bound and cross-sectional. A more in-depth study is required to form a clearer picture of the situation in the rural and remote areas with longitudinal follow up. There is also a need for further research targeting more vulnerable groups like children, adolescents and women.

## CONCLUSIONS

There was prevalence of interpersonal violence during the COVID-19 lockdown in Nepal. This is a double injustice in that victims not only have to face fear regarding the pandemic but also domestic violence. The stay at home orders and lack of mobility options traps victims in their own home with limited options to seek help. Identification of vulnerable groups and proper management of interpersonal violence has to be among the priority given its high prevalence, pervasive nature and almost unanimous expert consensus on its increase during periods of heightened stress. A solution to the problem of underreporting might be longitudinal studies, which have a higher yield of findings. Identifying the gender, age bracket, living arrangement and educational qualifications of the victims of interpersonal violence may be the first step in dealing with this problem.
